# Evaluation of auditory perception development in neonates by quantitative electroencephalography and auditory event-related potentials

**DOI:** 10.1371/journal.pone.0183728

**Published:** 2017-09-14

**Authors:** Qinfen Zhang, Qirui Cheng, Hongxin Li, Xuan Dong, Wenjuan Tu

**Affiliations:** 1 Changzhou Children's Hospital,Changzhou,Jiangsu,PR China; 2 Nanjing Children's Hospital, Nanjing, Jiangsu, PR China; University of Electronic Science and Technology of China, CHINA

## Abstract

**Objective:**

The present study was performed to investigate neonatal auditory perception function by quantitative electroencephalography (QEEG) and auditory event-related potentials (aERPs) and identify the characteristics of auditory perception development in newborns.

**Methods:**

Fifty-three normal full-term neonates were divided into three groups according their age in days. An auditory oddball paradigm was used. QEEG (resting state and task state) and aERPs were performed. EEG δ power in the resting and task states and at different ages was respectively analyzed. The N2 area and latency of aERPs at different ages were also compared.

**Results:**

The four main findings of this study are as follows. First, the increase in the EEG δ power was significantly greater in the task than resting state in Group 3 at the Fz lead (t = −3.371, P = 0.004) and in Groups 2 and 3 at the Cz lead (Group 2: t = −3.149, P = 0.005; Group 3: t = −3.609, P = 0.002). Second, the δ power gradually increased from 1 to 10 days of age (Group 1), peaked at 11 to 20 days (Group 2), and gradually decreased from 21 to 28 days (Group 3). The data in the Fz lead during the task state and in the Cz lead during the resting and task states were statistically significant (F = 5.875, P = 0.005; F = 5.523, P = 0.007; and F = 5.402, P = 0.008, respectively). Third, the N2 area significantly increased with age by presentation of target stimuli (F = 5.26, P = 0.01). The N2 area increased most significantly from 21 to 28 days (Group 3). Finally, the N2 latency significantly decreased with age (Fz lead: F = 4.66, P = 0.023; Cz lead: F = 7.18, P = 0.005). The N2 latency decreased most significantly from 11 to 20 days of age (Group 2).

**Conclusion:**

Rapid cognitive development occurs during the neonatal period. In the first several days after birth, the EEG δ power and N2 area manifested the characteristic performance of identifying task information. QEEG and aERP measurement can be used as objective indices with which to evaluate auditory perception development in neonates.

## 1. Introduction

Cognitive function refers to the process of acquiring, coding, manipulating, extracting, and using sensory input information when recognizing objective items. Cognitive function represents the internal psychological process between input and output. In 1950, the Swiss psychologist Jean Piaget developed a cognitive development theory that remains the most comprehensive model of infant cognition. He proposed that within 1 month after birth, an infant can initiate the acceptance of a variety of sensory stimulations to formulate a reflex response, forming the cornerstone of future cognitive development. The neonatal stage is the most prosperous and critical period in brain growth and development[1].The study of brain function in newborns has thus attracted much attention.

Research of cognitive function has recently made great progress largely because of advancements in research methods. Newborns have limited motor function and no language function. Electroencephalography (EEG) is a routine examination tool with which to study neonatal brain function because of its non invasiveness and maneuverability. EEG is more sensitive than morphological clinical indicators in its reflection of brain function, showing the projection of neuronal post synaptic potentials to macroscopic potentials of the scalp. Quantitative EEG (QEEG) is a quantitative measure of the original EEG data and overcomes the subjective interference of traditional visual analysis. It is a computer-based technology that is used to calculate and display EEG signals in the time and frequency domains. The core technology of QEEG is power spectrum analysis. The power spectrum clearly displays the distributions of and changes in α-, β-, θ-, and δ-band brain waves. The δ wave forms the main brain activity rhythm in the neonatal period, reflecting the capacity for signal detection and decision-making[2]. The EEG δ power can be used as an electrophysiological indicator of cortical functional maturation during EEG examination[3].

QEEG has been used for clinical diagnosis and research in the fields of psychosis, epilepsy, and quantitative drug analysis. It also has the potential for wide application in various physiological and pathological states of brain function as well as in the fields of psychology and pharmacology. However, the use of QEEG in research of pediatric cognitive function, especially in the neonatal stage, is new.

Event-related potentials (ERPs) are associated with sensory, motor, or cognitive events with a high temporal resolution[4]. ERPs are electrophysiological signals induced by auditory, visual, and somatosensory stimulation. ERP measurement is currently considered the most effective tool for assessing infant brain function[5]. Auditory ERPs (aERPs) are produced by the cortex during the processes of consciousness, cognition, memory, and judgment of auditory stimuli.

Auditory perception completely develops in the early stages of pregnancy (27–29 weeks)[6]. The fetus has an auditory stimulating response at 27 weeks of gestation[7]. Audition is an element of recognition and analysis at the preconsciousness level in the cerebral cortex. It also reflects information processing and other cognitive functions of the cerebral cortex in different development stages. Therefore, the neonate can use an auditory paradigm to evoke aERPs. Neonatal studies involving auditory tasks such as articulation and prosody[8] and oddball stimulus sequences[9] have been carried out in recent years, providing evidence of the aERP characteristics of newborns under cognitive tasks. Because measurement of aERPs is easy to perform, noninvasive, and does not require active cooperation, it is suitable for the evaluation of cognitive brain function in newborns, who have no language function and limited motor function[10,11]. However, aERP technology is infrequently used in neonatal research and even less frequently in research of newborns’ cognitive development.

In the present study, quantitative and noninvasive QEEG and aERPs were used to measure the cognitive function of neonates to determine the characteristics and regularity of neonatal cognitive development.

## 2. Methods

### 2.1 Participants

Sixty full-term neonates were recruited from the Department of Neonate, Changzhou Children’s Hospital (affiliated with Nantong University, China) from March 2014 to December 2015. The inclusion criteria were gestational age (40 ± 1 w), Apgar score of ≥8 at 1 and 5 min, absence of brain injury during the perinatal period, neonatal behavioral neurological assessment score of >37, both ears passing hearing screening, stable vital signs, and no obvious organic diseases. All of these criteria were evaluated by professionals in the Department of Neonate. Neonates with one or more of the following conditions were excluded: neonatal encephalopathy, intracranial hemorrhage, severe hyperbilirubinemia, craniofacial malformation, congenital brain abnormalities, genetic or metabolic diseases, test tube babies, and twins.

Seven neonates were excluded because of artifacts. The included neonates were assigned to three age groups: 1–10 days after birth (Group 1, n = 16), 11–20 days after birth (Group 2, n = 20), and 21–28 days after birth (Group 3, n = 17) (Table 1).

**Table 1:** Baseline characteristics of the study population.

Newborn Characteristic	Group 1	Group 2	Group 3	P-value
**Sex-male n(%)**	7(43.8)	11(55)	8(47.1)	0.79
**Birth gestation age-weeks(SD)**	40.00(0.68)	40.10(0.68)	40.20(0.56)	0.72
**Birth weight-kg(SD)**	3.44(0.30)	3.55(0.26)	3.49(0.27)	0.45
**Age at visit-days(SD)**	5.75(2.35)	15.45(2.44)	23.23(2.07)	<0.01
**Weight at visit-kg(SD)**	3.56(0.25)	3.78(0.23)	3.75(0.17)	<0.01
**1 min Apgar(SD)**	9.15(0.81)	9.00(0.86)	8.88(0.78)	0.69
**5 min Apgar(SD)**	9.31(0.70)	9.25(0.85)	9.35(0.70)	0.92
**NBNA score(SD)**	38.94(0.85)	38.90(0.79)	38.92(0.78)	0.99

Experimental aERP detection is a noninvasive test, and its application was approved by the ethics committee of Changzhou Children’s Hospital. The participants’ families gave informed consent according to the requirements of the ethics committee.

### 2.2 Stimuli and procedures

The awake state involves the resting state (awake and quiet without external stimulus tasks) and the task state (presence of auditory stimuli). The neonates in the task state were presented with an auditory oddball paradigm. Target stimuli were 2000-Hz tone bursts and non-target stimuli were 1000-Hz tone bursts; each lasted for 100 ms with 1500-ms stimulus intervals. The target or non-target stimuli appeared randomly. The experimental design consisted of 3 blocks of 330 stimuli for each infant, for a total of 990 stimuli. The frequency was 10% for target stimuli and 90% for non-target stimuli. All stimuli were produced at a sound pressure level of 50 dB. The stimuli were controlled by E-Prime 2.0 software (Psychology Software Tools, Pittsburgh, PA, USA).

The laboratory was sound-insulated and maintained at a temperature of 24°C to 26°C. The neonates were positioned on a comfortable bed with a speaker placed 15 cm away from each ear. EEG was performed at various electrodes (Fz, Cz, F3, F4, C3, and C4) and referenced to the bilateral earlobes according to the International 10–20 system. Only these sites were used to ensure that the smallest possible number of electrodes was placed on the scalp given the clinical condition of the newborns. Sound stimuli were then administered. No sedatives were used on any participants. After being fed, the newborns were satisfied and quiet. We gently wrapped the newborns’ hands and feet in a quilt to prevent limb movement.

First, the participants underwent EEG in the resting state to obtain the resting EEG power. EEG in the task state was then performed to obtain the task state EEG power and aERPs.

### 2.3 ERP measurement and data processing

A digital 32-channel EEG recording apparatus was used (Stellate Systems Inc., Quebec, Canada). It had an amplifier bandpass filter of 0.53 Hz and sampling rate of 500 Hz. Resting EEG and task state EEG were recorded for about 35 min.

BESA software (MEGIS Software Co., Munich, Germany) was used to analyze the EEG data. Ninety 5-s EEG fragments were randomly selected in each state. The EEG power values of δ, θ, α, β, and γ were obtained by fast Fourier transform. The power of the frequency band (0.5–4.0 Hz) of the Fz and Cz leads in the resting and task states were then measured.

ERP data processing was also performed using BASA software. N2 latencies and areas were measured for target stimuli at the Cz and Fz leads. N2 latencies were measured as the length of time between the start of the stimulus to the wave ridge. If two or more peaks existed, then the crossing point of two lines extrapolated from the ascending and descending portions of the waveform was taken as the vertex of the peak. The N2 area refers to the area between the N2 wave and baseline. If an N2 waveform drifted or was difficult to determine, then the start time of N2 for the average ERP waveforms was used as the time window for measurement. The area between the curves and baseline within this time window was measured as the N2 area.

### 2.4 Statistical analysis

Analyses were performed using SPSS for Windows, version 19.0 (IBM Corp., Armonk, NY). The level of statistical significance was P < 0.05. The paired-samples t test was conducted to compare the EEG δ power in the resting state with that in the task state. One-way analysis of variance was used to analyze the EEG δ power and ERP N2 area and latency data, respectively, at different days of age.

## 3. Results

### 3.1 Neonatal EEG power

#### 3.1.1 Comparison of EEG power between resting and task states at Fz and Cz leads in neonates

**Fig 1:**
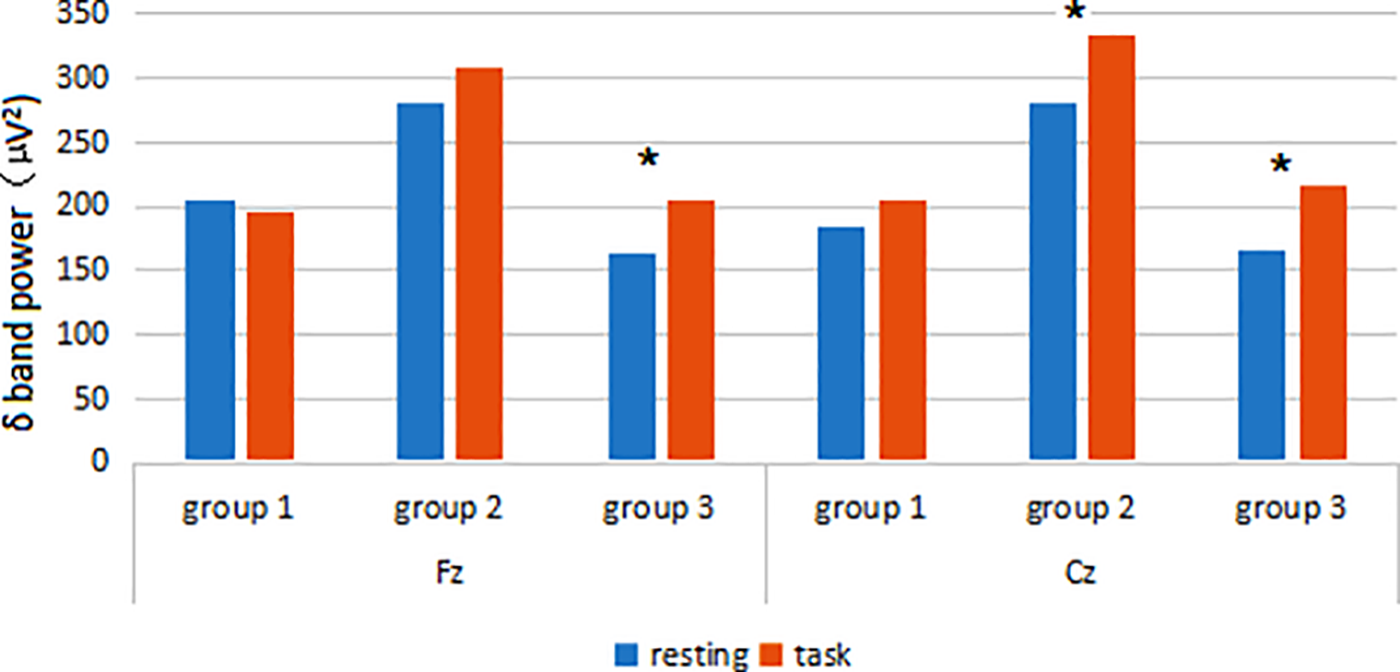
Comparison of EEG δ power between resting and task states at Fz and Cz leads.

At the Fz lead, Group 3 had a significantly higher EEG δ power in the task state than in the resting state (resting: 162.23 ± 63.10 μV^2^ vs. task: 204.07 ± 91.72 μV^2^, t = −3.371, P = 0.004). No significant differences were observed between the two states in Group 1 (resting: 205.01 ± 133.69 μV^2^ vs. task: 195.71 ± 109.64 μV^2^, t = 0.256, P = 0.802) or Group 2 (resting: 280.06 ± 129.95 μV^2^ vs. task: 309.21 ± 129.66 μV^2^, t = −1.727, P = 0.100) (Fig 1).

At the Cz lead, significant differences were observed in the EEG δ power between the two states in both Group 2 (resting: 281.41 ± 121.73 μV^2^ vs. task: 334.42 ± 135.95 μV^2^, t = −3.149, P = 0.005) and Group 3 (resting: 166.39 ± 84.43 μV^2^ vs. task: 216.64 ± 89.85 μV^2^, t = −3.609, P = 0.002). No significant differences were observed in Group 1 (resting: 184.38 ± 129.96 μV^2^ vs. task: 203.68 ± 165.96 μV^2^, t = −0.409, P = 0.688) (Fig 1).

#### 3.1.2 Comparison of EEG power development at different days of age in both resting and task states in neonates

**Fig 2:**
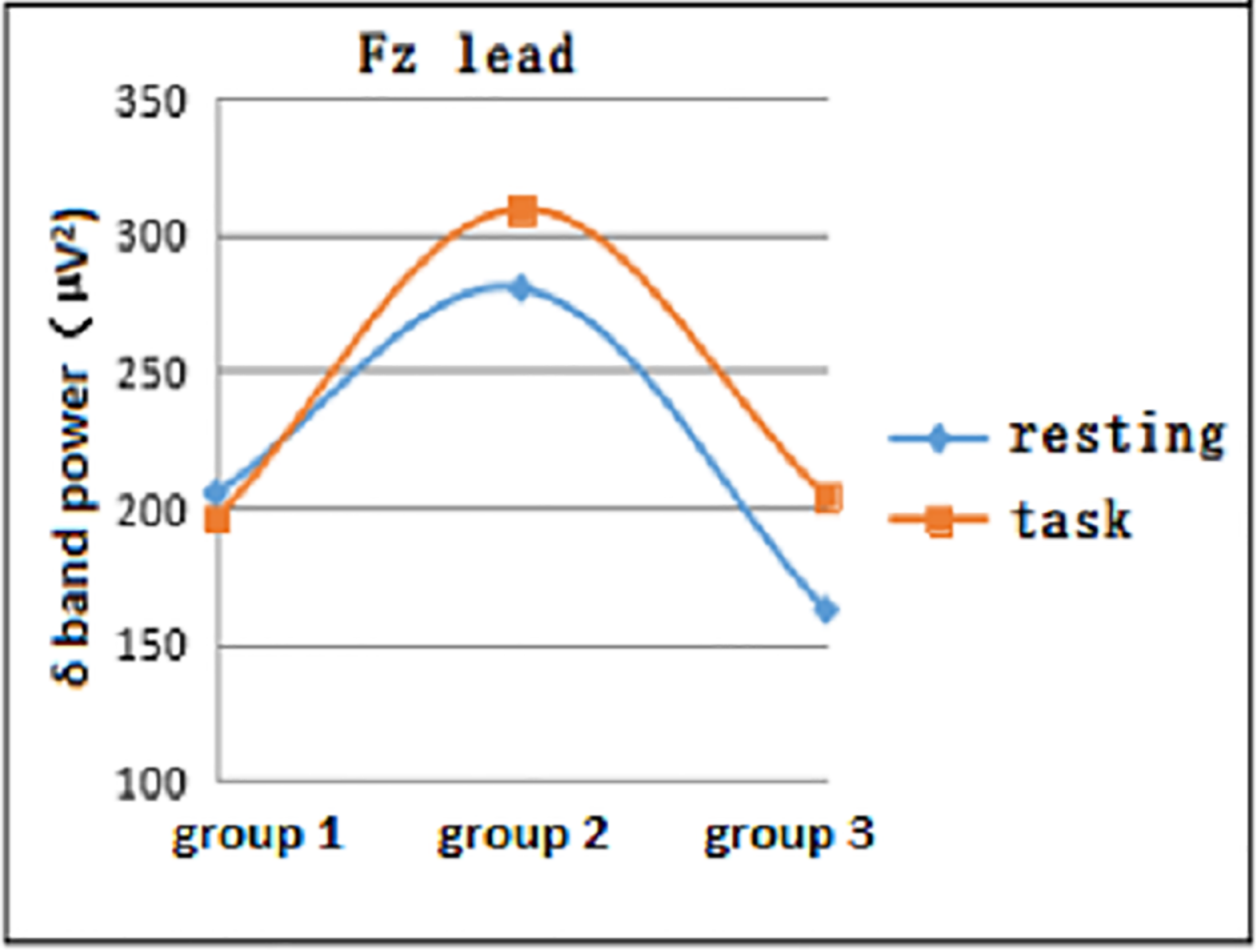
EEG δ power of resting and task states in the three groups at the Fz lead.

**Fig 3:**
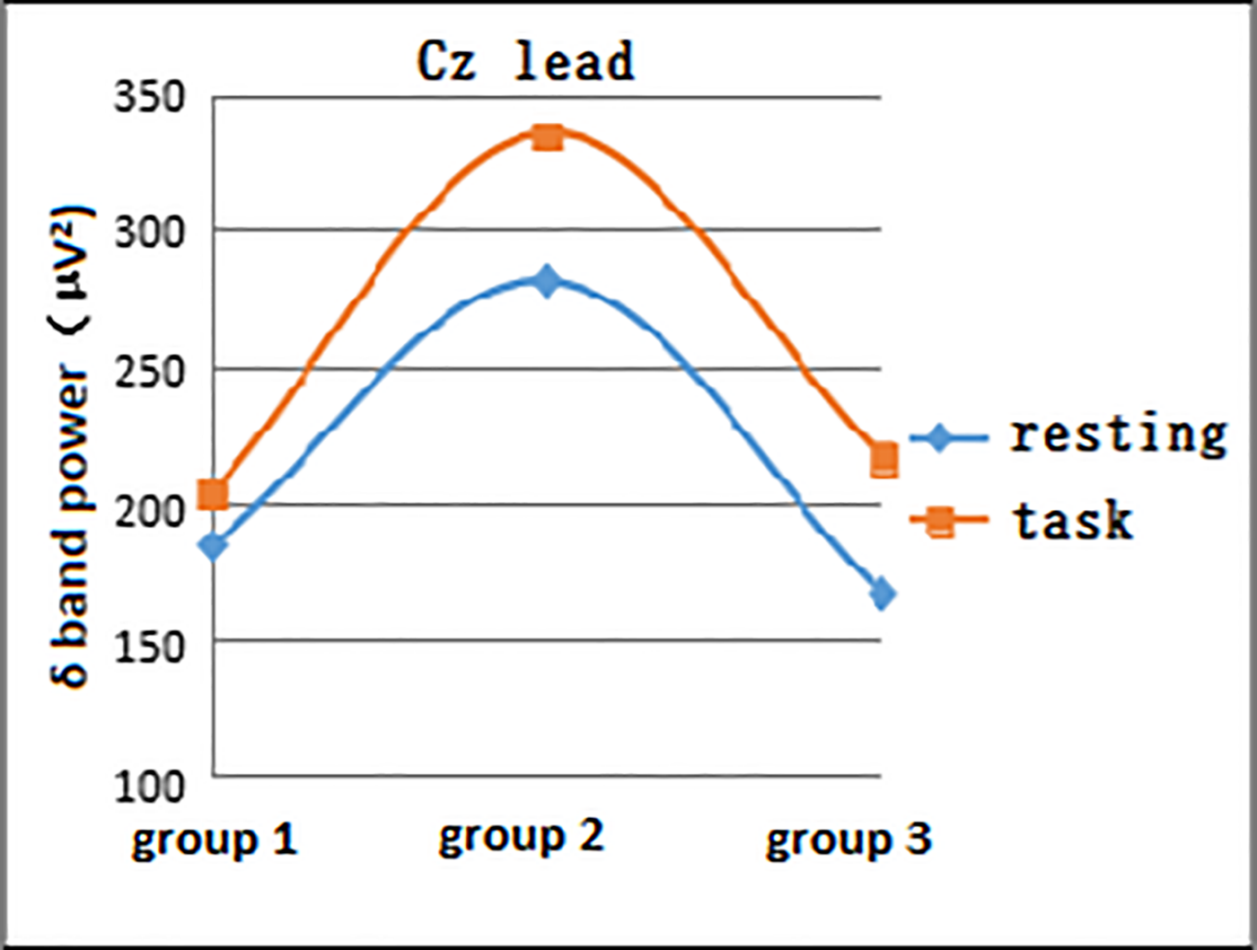
EEG δ power of resting and task states in the three groups at the Cz lead.

In both the resting and task states, the EEG δ power at different days of age gradually increased in the 1- to 10-day age group (Group 1), peaking in the 11- to 20-day age group (Group 2) and gradually decreasing in the 21- to 28-day age group (Group 3). The data in the Fz lead task status and Cz lead resting and task states were statistically significant (F = 5.875, P = 0.005; F = 5.523, P = 0.007; and F = 5.402, P = 0.008, respectively) (Figs 2 and 3).

### 3.2 Neonatal ERP study

**Fig 4:**
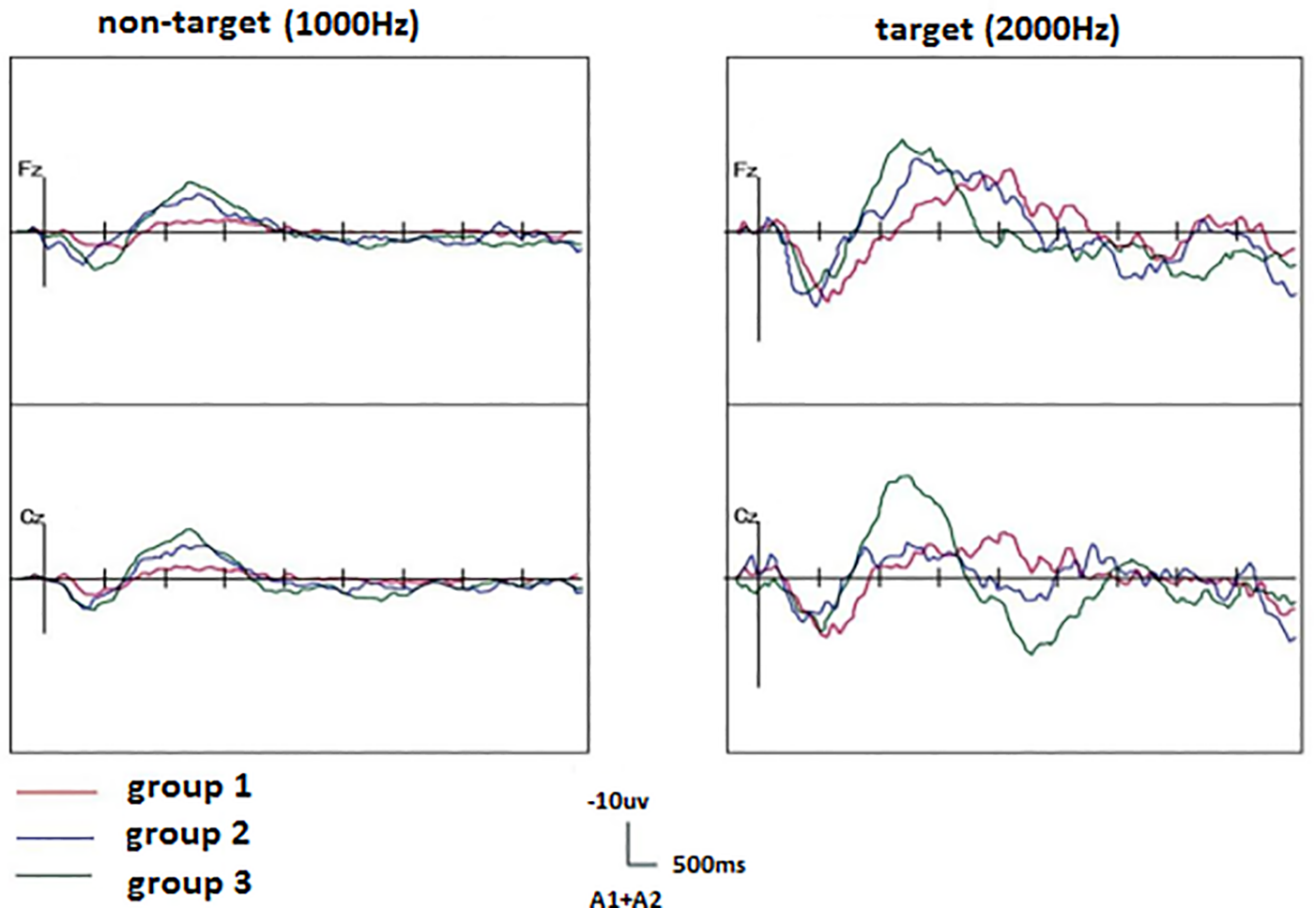
Neonatal grand averages of ERP at the Fz and Cz leads.

The grand averages of the neonatal ERP waveforms by target stimuli started from relatively irregular flat-bottomed troughs, yielding curves with a jagged appearance. The N2 waves exhibited a complex of several flat-bottomed peaks from 1 to 10 days after birth (Group 1), whereas, relatively regular, steep-sided ripples tended to present as single waves at 20 to 28 days after birth (Group 3) (Fig 4).

#### 3.2.1 Comparison of N2 area in the three groups by target and non-target stimuli

**Fig 5:**
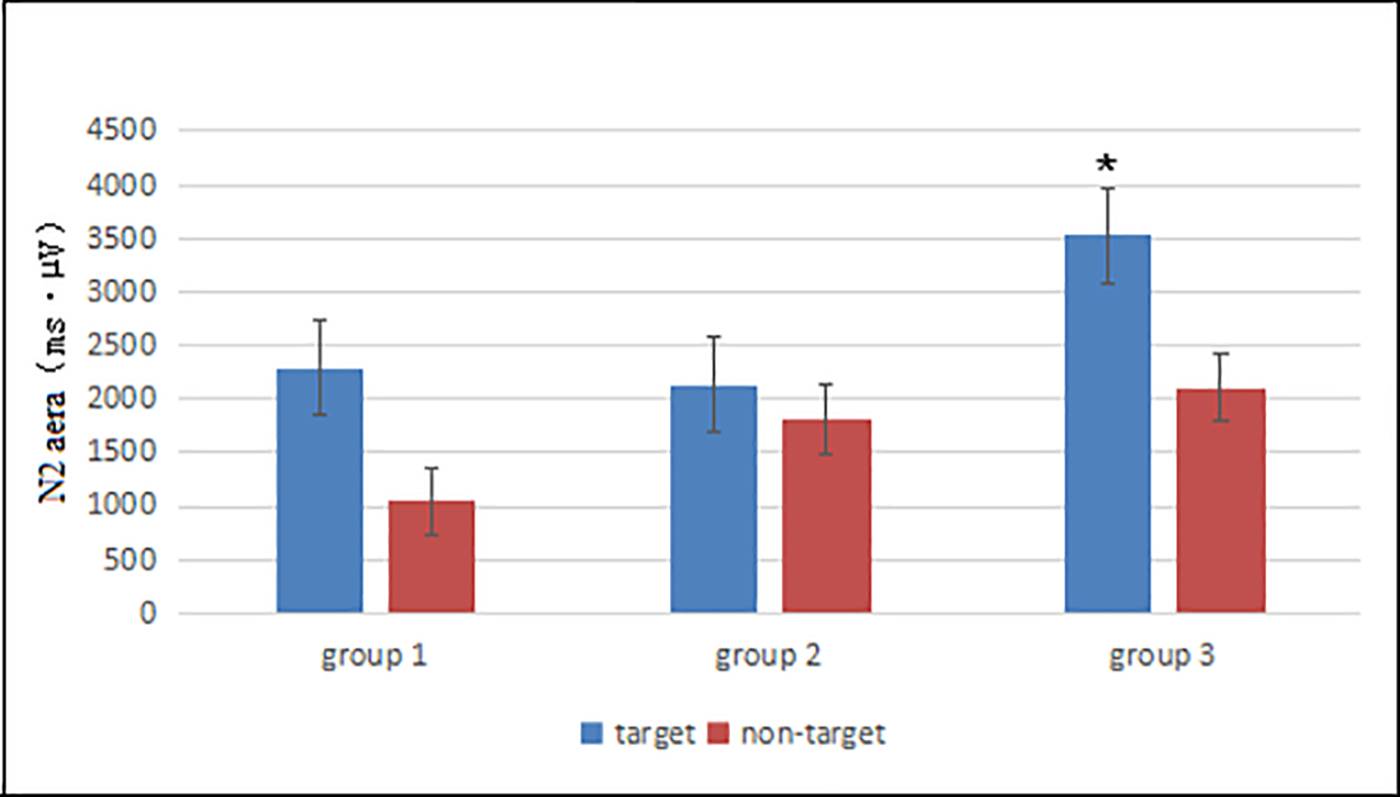
Comparison of N2 wave area in the three groups by target and non-target stimuli. *P < 0.05.

With the target stimuli, the N2 area increased significantly according to the days of age (Group 1: 2281.3 ± 1063.3 ms·μV, Group 2: 2125.4 ± 993.0 ms·μV, Group 3: 3526.7 ± 994.4 ms·μV, F = 5.26, P = 0.01). There were significant differences between Groups 1 and 3 (P = 0.015) and between Groups 2 and 3 (P = 0.006). There was no significant difference between Groups 1 and 2 (P = 0.74). With the non-target stimuli, no significant difference was observed between the N2 area and the days of age (Group 1: 1044.31 ± 821.29 ms·μV, Group 2: 1813.16 ± 1238.59 ms·μV, Group 3: 2109.11 ± 2878.37 ms·μV, F = 0.37, P = 0.49). These findings suggest that with increasing neonatal age, the N2 area gradually increases with target stimulation. The N2 area increased most significantly at 21 to 28 days (Group 3) (Fig 5).

#### 3.2.2 Comparison of N2 latency in the three groups

**Fig 6:**
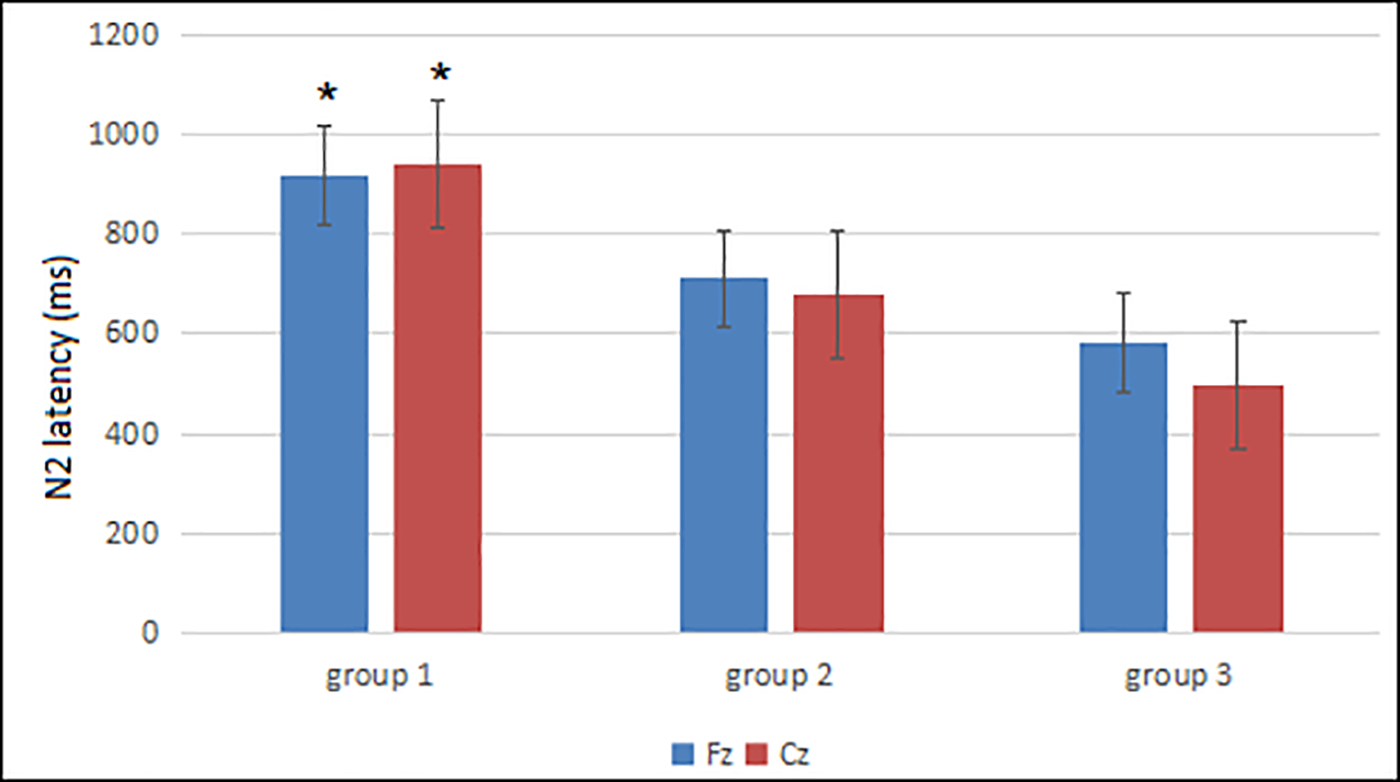
Comparison of N2 latency at Fz and Cz leads in the three groups. *P < 0.05.

The N2 latency in the three groups significantly decreased with age at the Fz lead (Group 1: 919.25 ± 191.96 ms, Group 2: 711.75 ± 166.67 ms, Group 3: 582.33 ± 276.52 ms, F = 4.66, P = 0.023) and Cz lead (Group 1: 940.75 ± 285.31 ms, Group 2: 678.75 ± 155.92 ms, Group 3: 497.67 ± 192.33 ms, F = 7.18, P = 0.005). There was a significant difference between Groups 1 and 2 (Fz: P = 0.037, Cz: P = 0.039) and between Groups 1 and 3 (Fz: P = 0.019, Cz: P = 0.007). No significant difference was observed between Groups 2 and 3 (Fz: P = 0.296, Cz: P = 0.075). These findings suggest that the N2 latency shortened with increased neonatal age. The N2 latency decreased most significantly at 11 to 20 days of age (Group 2) (Fig 6).

## 4. Discussion

QEEG can directly and objectively display brain function. It is a scientific tool with which to study the characteristics of cognitive development in neonates. Auditory ERPs reflect the brain’s attention to processing and resolution of sound stimuli. Additionally, measurement of aERPs is an objective, quantitative, and noninvasive technique for evaluation of cognitive function during early life and can be used to test neonatal cognitive ability[6]. N2 waves, an endogenous component of ERPs that relate to higher brain functions including template matching, alertness, judgment, behavior, conflict monitoring, and cognitive control, primarily reflect the psychological process of identifying target stimuli and are closely related to cognitive processes[11,12].

### 4.1 Neonatal EEG power in resting and task states and its development at different ages

Bell and Wolfe[13] found that children’s EEG power exhibited task-related localized changes during memory tasks. Cuevas et al.[14] studied 5- to 10-month-old infants during different tasks and found that the EEG power of working memory increased. Chipaux[15] reported that stimulation by environmental sounds can lead premature infants to produce temporal δ brush activity.

In the present study, we found that the EEG δ power in the task state was significantly higher than that in the resting state, and the difference gradually increased with age, becoming statistically significant at 20 to 28 days. These findings indicate that the δ response significantly increased during the presentation of the cognitive stimuli in the auditory oddball paradigm and reflected the newborn’s resource allocation and energy input in cognitive activities. When the neonates noticed the stimuli of the auditory task, a higher number of neurons responsible for information processing were activated, and the activity levels of these neurons increased. The EEG power was then significantly improved. This process showed an increased development of responses to external sensory stimuli[16]. Energy related to judgment and processing of information gradually increased with the development of the neonates’ synapses and myelin.

Sankupellay et al.[17] considered that the sleep EEG power of 2-year-old children increased with myelination formation. Lippé et al.[18] found that from 1 month to 5 years of age, the spectral activity of the EEG signal gradually changed from θ to α by processing of auditory and visual stimuli. Niemarkt et al.[19] reported that the EEG activity increased with age during the neonatal period and that the EEG spectrum showed a significant change from low to high frequency.

The present study revealed that the EEG δ power gradually increased from 1 to 10 days of age and peaked at 11 to 20 days with the emergence of θ waves; the EEG δ power then gradually decreased from 21 to 28 days. At the time of birth, the number of nerve cells has reached the adult level; however, the number of synaptic connections is still low. As the number of synapses increases, myelination gradually matures and the neural circuits of information transmission and processing become more stable. The ability to process cortical information gradually increases. As the brain energy grows, the EEG rhythm changes from low-frequency δ-waves to high-frequency θ-waves. Thus, the neonatal EEG δ power tends to first increase and then decrease with age. This study also showed that the EEG δ power was highest at 11 to 20 days of age. We believe that the first 11 to 20 days after birth may be the most critical period during which the neonate accepts stimulus signals and responds. This coincides with the EEG activity pattern, which changes from discontinuous to triggering to continuous and more synchronized at the end of the second week after birth[20]. These changes in the EEG power reflect the changes in brain function during the neonatal period and are closely related to cognitive development in neonates.

### 4.2 ERP in neonates at different days of age after birth

The ERP wave area represents the sum of the biological potential discharged by cerebral neurons in units of time and is considered to represent the energy of the brain[21]. The ERP area is thought to be consistent with the ERP amplitude. Lamm et al.[22] studied ERPs in children aged 7 to 16 years and found that the N2 amplitude gradually decreased with age. In our preliminary study involving 1113 normal children aged 4 to 12 years, Dong et al.[23] found that the P300 latency was negatively correlated with age.

Our findings demonstrate that the N2 area gradually increases during the neonatal period. We consider this to be related to the characteristics of cortical development because the cerebral cortex is the largest and most complex structural component of the brain. At birth, neonates possess normal gyri, but these gyri are superficial[24]. During the process of constant deepening of the gyri, the cortical area continuously increases. In addition, the cerebral cortex contains many neurons and synapses, which constitute the neural circuitry. At birth, the number of neurons has reached the level of adults, but few synapses are present. There is a long path to accomplishment of construction of the neural circuits responsible for information transmission and processing. Although some neural circuits have been established, they are not firm. With an increasing number of neurons, the somas enlarge and synapses form, stabilize, and mature. This contributes to differentiation of the brain, formation of a complex neural network, and final maturation of the brain. During the neonatal period, the N2 area gradually increases, indicating strengthening of cortical function and neural networks and the development of neonatal cognitive processing.

The results of this study demonstrate that N2 latency gradually shortens with age during the neonatal period. We considered that this possibly occurred because of myelinization, the process by which axons are enveloped in a myelin sheath, which serves to accelerate neuronal transmission along nerve fibers and ensure directional transmission. At birth, myelination has not yet been implemented; thus, the bioelectrical conduction velocity is slow, it takes a long time for infants to judge stimuli and process signal information, and N2 latency is prolonged. With increasing age after birth, the degree of myelination increases and segmental structures transmit nerve impulse rapidly and precisely in a manner of leaps, contributing to constantly accelerating information processing. Hence, the N2 latency tends to gradually shorten.

Latency represents the speed at which the brain categorizes and recognizes external stimuli and is a hallmark of the information processing time[25]. Most studies of N2 latency have been performed in preschool- or school-aged children, and the conclusions are controversial. In one aERP study conducted by Hövel et al.[26], the N2 latency obviously shortened with increasing age of infants born at <32 weeks of gestation and at full term. Cunningham et al.[27] reported shortened N2 latency in 5- to 6-year-old children; however, Wunderlich et al.[28] concluded that there was no significant difference in N2 latency in children aged 1 to 6 years.

### 4.3 Study of EEG power and ERPs in newborns

The present study revealed that the ERP N2 area gradually increased with days of age and peaked at 20 to 28 days; the difference in the EEG δ power between the task state and resting state also peaked at 20 to 28 days of age. Moreover, we found that the N2 latency shortened with age; this became most obvious at 11 to 20 days. The EEG δ power also peaked at 11 to 20 days. All of these changes reflect the synchronization of maturity on the development of cognitive function and brain function. The first 11 to 20 days after birth may be the most critical period during which the infant accept stimulus signals and responds; it may also be a special period of cognitive and brain function development. Further research is needed to test this hypothesis.

## 5. Limitations

We only collected aERP data of 53 neonates because of poor cooperation, and the analysis of EEG power and aERP development characteristics was limited. Because neonatal aERP research is still in its infancy, the N2 component was evaluated in this study. However, the other components of neonates need to be further explored.

## Supporting information

S1 Data Age of check, Apgar score, born weight, gestational age, NBNA score. (XLS)
