# Correction: Evaluation of auditory perception development in neonates by quantitative electroencephalography and auditory event-related potentials

**DOI:** 10.1371/journal.pone.0263361

**Published:** 2022-01-26

**Authors:** Qinfen Zhang, Qirui Cheng, Hongxin Li, Xuan Dong, Wenjuan Tu

There is an error in affiliation 2 for author Qirui Cheng. The correct affiliation 2 is: Children’s Hospital of Nanjing Medical University, Nanjing, Jiangsu, PR China.
